# Quality of life among adolescents with idiopathic scoliosis in Tunisia

**DOI:** 10.11604/pamj.2023.45.27.38575

**Published:** 2023-05-08

**Authors:** Aymen Haj Salah, Soumaya Arem, Manel Ben Fredj, Meriem Rekik, Ikram Haddada, Bessem Krifa, Mouna Sghir, Wassiaa Kessomtini

**Affiliations:** 1 Department of Physical Medicine and Rehabilitation, University Hospital Taher Sfar, Mahdia, Tunisia,; 2 Faculty of Medicine of Monastir, University of Monastir, Monastir, Tunisia,; 3 Department of Epidemiology and Preventive Medicine, University Hospital Fattouma Bourguiba, Monastir, Tunisia

**Keywords:** Scoliosis, adolescent, quality of life, rehabilitation, Tunisia

## Abstract

**Introduction:**

our study aimed to assess the quality of life (QOL) among adolescents with adolescent idiopathic scoliosis (AIS) receiving nonoperative treatment, and to identify the demographic and clinical factors associated with poor QOL.

**Methods:**

this is a cross-sectional study. We included adolescents followed in the Department of Physical Medicine and Rehabilitation at Taher Sfar Hospital (Mahdia - Tunisia). The Quality-of-Life Profile for Spine Deformities (QLPSD), the Scoliosis Research Society 22 questionnaire (SRS-22) and the visual analogue scale objectifying the QOL (EVA QOL) were used. Correlations between the QOL domains and selected characteristics were performed.

**Results:**

a total of 48 adolescents with AIS were included, with a mean age of 14.2 ± 2.1 years and a sex ratio (M/F) of 0.77. Adolescents who underwent rehabilitation treatment only had significantly better quality of life (QOL) scores than those with braces, as measured by three scales. Among brace wearers, we found a correlation between QOL and the degree of correction achieved by the brace, as measured by the EVA-QOL. We observed significant relationships between psychosocial status and age, correction angle, and treatment duration among braced patients, as measured by the QLPSD. Additionally, we found that dorsal flexibility was correlated with the correction angle and the treatment duration. According to the SRS-22, the overall QOL score of braced adolescents was significantly correlated with the correction obtained by the brace.

**Conclusion:**

wearing a brace in adolescent with AIS leads to a significant decrease in QOL according to the three QOL assessment scales.

## Introduction

Scoliosis is the most common spinal disorder in children and adolescent [1,2]. It is a three-dimensional deformation of the spine, characterized by a lateral curvature of the latter greater than 10° in the frontal plane on a spine radiograph [3]. Adolescent idiopathic scoliosis (AIS) accounts for approximately 90% of idiopathic scoliosis cases [4]. Current data showed an overall AIS prevalence of 0.47 to 5.2% [3,5]. The severity and progression of scoliosis is greater in girls than in boys [3]. This deformity can be accompanied by spinal pain, lung damage, and psychological disorders [6-9]. Orthopedic treatment is not intended to correct the curvature, but rather to slow or stop the progression of the curve [10]. However, the results obtained were very disparate depending on the studies [11]. The trunk orthosis models available for this disease are numerous. The brace is indicated for lumbar or thoracolumbar scoliosis. In most cases, it allows for a good reduction. Its implementation is simpler, and the tolerance is better than that of an initial treatment with plaster. Although the brace is an essential treatment for AIS, it can have a negative effect on the physical and psychological levels, particularly in adolescents, by considerably modifying their rhythm of life [11]. Nevertheless, the impact of wearing a brace on the quality of life (QOL) of the adolescent is still poorly evaluated in developing countries. Our study aimed to measure the QOL in Tunisian adolescents with AIS under nonoperative treatment. We also aimed to identify the demographic and clinical factors associated with poor QOL in braced patients.

## Methods

The guidelines of Strengthening the Reporting of Observational Studies in Epidemiology (STROBE) statement were followed [12].

**Study design:** we carried out a cross-sectional study.

**Setting:** this study was conducted in the Department of Physical Medicine and Rehabilitation at the University Hospital Taher Sfar (Mahdia-Tunisia), during a period of six months from September 2016 to February 2017.

**Study population:** adolescents diagnosed with AIS and attending the department during the study period were recruited.

**Inclusion criteria:** patients diagnosed with AIS and aged between 10 and 18 years old; patients proposed for rehabilitation only or associated with bracing; being treated for at least two months; having no history of spinal surgical treatment.

**Exclusion criteria:** non-idiopathic scoliosis; other comorbidities; no consent provided by the participant or his parents to be enrolled in the study.

**Variables:** demographic variables such as age and gender were collected. We collected the following clinical characteristics: curvature type (thoracic, lumbar, thoracolumbar), treatment type, treatment duration, Cobb angle, Risser index and percentage of correction. The Cobb angle is a measurement used to determine the degree of spinal curvature in individuals with scoliosis. To measure the Cobb angle, X-rays of the entire spine are taken in a standing position. Then we Identify the most tilted vertebrae at the top and bottom of the curve and we draw a line perpendicular to the top of the upper tilted vertebrae and a line perpendicular to the bottom of the lower tilted vertebrae. We measure the angle formed by the intersection of these two lines using a protractor [13]. The Risser index divided the steps of ossification and fusion of the iliac apophysis into six stages (Risser Stages 0-5) describing the advancement toward skeletal maturity. Stage 0 describes an X-ray on which no ossification center is seen in the apophysis, whereas stage 5 represents complete ossification and fusion of the iliac apophysis. Two slightly different versions of the Risser system are in use; the differences concern stages 2 to 4 [14]. The United States Risser staging system divides the excursion of the apophysis into quarters of the iliac crest beginning anterolaterally and progressing poster medially, whereas the French Risser staging system divides the excursion of the apophysis into thirds (Stages 1-3), with Stage 4 representing complete ossification and initiation of apophyseal fusion. In the current study, we have used the French Risser staging system.

The percentage of correction is measured by comparing the Cobb angle measurement from the X-ray taken while the patient is wearing the brace to the initial Cobb angle measurement taken before the patient started wearing the brace (before and after treatment). We calculate the percentage of correction by subtracting the brace Cobb angle from the initial Cobb angle and dividing the difference by the initial Cobb angle. Then, multiply the result by 100 to express the percentage. According to treatment type, two groups were identified: a braced group (G1) and an unbraced group (G2). The two groups benefited from rehabilitation with 3 sessions per week for at least two months. For adolescents who underwent rehabilitation only, the indications were Cobb angle < 20° at the first consultation, corset being made, nonprogressive scoliosis or Risser > 4. All braced adolescents wore the Cheneau brace. All participants completed the questionnaires measuring the QOL.

**Measuring instruments:** the QOL was assessed using Climent's “the Quality-of-Life Profile for Spine Deformities” (QLPSD) scale, the Scoliosis Research Society 22 questionnaire (SRS-22) and by a visual analogue scale objectifying the QOL (EVA-QOL). The QLPSD is a scale that assesses the QOL of adolescents or preadolescents aged from 10 to 20 years old with a spinal deformity, on a growing spine [15]. This scale includes five domains evaluating respectively the psychosocial state, sleep disorders, back pain, body image and dorsal flexibility (flexibility of the trunk). This questionnaire contains 21 items evaluated from 1 to 5 points, 5 corresponding to the worst experience (totally agree) and 1 to the best experience (totally disagree). A high score means a poor QOL. The SRS-22 is a questionnaire specific to spinal deformities. It has been validated in Arabic [16]. This questionnaire includes five domains evaluating respectively function, pain, body image, psychosocial state, and satisfaction with treatment. The sub scores for each domain were obtained by calculating the average of the corresponding items with a maximum score of 5. A low score means a poor QOL. We also used an EVA-QOL graded on a scale from 0 to 100 mm. A score of zero indicates a complete lack of embarrassment in daily life.

**Statistical analysis:** data entry was performed using Statistical Package for the Social Science Software (SPSS) 20.0. The qualitative variables were expressed in the form of effectives and percentages. The Kolmogorov-Smirnov test was used to test the distribution of quantitative variables (QV). Quantitative variables were expressed as means and standard deviation (SD). In univariate analysis, we used the chi-2 test for the comparison of the qualitative variables, the Student test for independent samples for the comparison of the quantitative variables. The Pearson correlation coefficients were also used. A significance level of less than 5% was retained for all statistical tests. A positive significant correlation (positive value of r with p<0.05) means a proportional relationship between the two QV. A negative significant correlation (negative value of r with p<0.05) means that the relationship between the two QV is inversely proportional.

**Ethical considerations:** this study was carried out in compliance with fundamental ethics principles. Patients were enrolled voluntarily. The objectives of the study were explained in a simple way to participants. Informed consent was taken from all adolescents and their legal guardians before inclusion in the study. Data confidentiality was guaranteed. A written approval from the Ethics Committee (Faculty of Medicine of Monastir) was obtained. The ethics committee approved the research protocol and the data collection methods. All participants completing the survey were not remunerated.

## Results

Forty-eight children were included in this study, with a mean age of 14.2 ± 2.1 years (10-18 years); and a sex ratio (M/F) of 0.77 (21/27). The M/F sex ratio in the braced group (G1) and in the unbraced group (G2) was 0.71 and 0.84, respectively. In terms of epidemiological data, the two populations were comparable. There was no significant difference for age and gender as well as for skeletal maturation (Risser index). The groups were significantly different (p < 0.001) for the Cobb angle, the percentage of correction obtained by the treatment and its duration. In most cases, scoliosis was thoracic or thoracolumbar. The characteristics of both groups were summarized in Table 1. The comparison of the QOL domains by treatment type was summarized in Table 2. According to the three scales, total scores were significantly better in adolescents treated only by rehabilitation than braced ones; with mean scores of 38.37±16.07, 39.95± 7.07 and 3.74±0.25 for EVA-QOL, QLPSD and SRS-22 respectively versus 55.70±17.60, 75.25±5.87 and 1.98±0.02 in the braced group.

**Table 1:** characteristics of study population

Variables	Patients with AIS treated by rehabilitation and brace (G1) (n=24)	Patients with AIS treated by rehabilitation-only(G2) (n=24)	p
**Gender: n (%)**			NS
Boys	11(48)	13(52)	
Girls	13(52)	11(48)	
Age (years):(mean±SD)	14.16±2.0	13.87±2.45	NS
Risser index:(mean±SD)	2.66±1.04	2.5±1.58	NS
Cobb angle (degree)(mean±SD)	30.66±5.75	8.91±3.11	<0.001*
Percentage of correction by the treatment (%): (mean±SD)	54±9.65	23±6.3	<0.001*
**Curvature type : n(%)**			
Thoracic	12(50)	8(33.3)	
Thoracolumbar	12(50)	12(50)	
Lumbar	0	4(16.7)	NS
Treatment duration (months)(mean±SD)	6.4±2.8	9.2±4.5	<0.001*

AIS : Adolescent Idiopathic Scoliosis, * : p<0.05 ; NS : nonsignificant

**Table 2:** quality of life in patients with IS according to three scales

Variables		Patients with AIS treated by rehabilitation and brace (G1)	Patients with AIS treated by rehabilitation only(G2)	p
**EVA-QOL** (mean±SD)		55.70±17.6	38.37±16.07	0,003*
**QLPSD** (mean±SD)	Psychosocial status	26.37±3.17	16.20±5.64	0,033*
	Sleep disorders	13.45±2.37	7.33±3.15	0,04*
	Back pain	10.87±1.56	6.20±1.81	0,002*
	Body image	14±1.74	6.66±2.01	0,001*
	Dorsal flexibility	10.54±1.66	3.54±1.31	0,001*
	Total	75.25±5.87	39.95±7.07	<0,001*
**SRS-22** (mean±SD)	Function	2.03±0.46	3.66±0.58	0,01*
	Pain	1.98±0.57	3.82±0.44	0,001*
	Body image	2.0±0,49	3,86±0,56	0,004*
	Psychosocial Status	2 .0±0,46	3,85±0,60	0,004*
	Satisfaction	1,87±0,43	3,68±0,49	0,001*
	Total	1,98±0,20	3,74 ±0,25	0,002*

IS : Adolescent Idiopathic Scoliosis, QLPSD : Quality of Life Profile for Spine Deformities" (QLPSD) scale, SRS-22: the Scoliosis Research Society 22 questionnaire; visual analogue scale objectifying the QOL (EVA-QOL) ; * : p<0,05 ; NS : nonsignificant

Similarly, scores by domains in QLPSD were higher in adolescents with brace than the other group according to the QLPSD, indicating a poor QOL in the adolescents treated with a brace. There was a significant difference between the two groups for psychosocial status, sleep disorders, back pain, dorsal flexibility, body image (p < 0.05) as well as the overall QLPSD score (p < 0.001). As for the SRS-22, the score of each QOL domain was significantly lower in the braced group than in the other group (p < 0.05). This indicated a poor QOL in adolescents treated by brace. The satisfaction domain was the most altered. There was a significant difference between the two groups for function, psychosocial status, pain, satisfaction, body image (p < 0.05) as well as the global SRS-22 score (p< 0.01). Among the braced group, the correlation coefficients between the QOL scores and the demographic and clinical characteristics are shown in Table 3. According to the EVA-QOL, we found a negative correlation of the QOL with the percentage of correction (EVA-QOL r=-0.3; p=0.014). In other words, as the percentage of correction increased, the QOL of the braced adolescents improved. No correlation was observed between QOL and other factors (age, Risser index or treatment duration).

**Table 3:** correlations between QOL domains and characteristics of braced patients

Variables		Age r(p)	Risser indexr(p)	Percentage of correction by brace : r(p)	Duration of treatment by brace: r(p)
**EVA QOL**		0.233 (NS)	0.091 (NS)	-0.3 (0.014*)	0.18 (NS)
**QLPSD**	Psychosocial status	0.493 (0.014*)	0.183(NS)	-0.23(0.014*)	-0.25 (0.031*)
	Sleep disorders	0.038(NS)	0.168 (NS)	0.057 (NS)	0.13(NS)
	Back pain	0.282 (NS)	-0.106(NS)	0.05 (NS)	-0.13 (NS)
	Body image	-0.12 (NS)	0.095 (NS)	-0.28 (0,01*)	-0.19 (NS)
	Dorsal flexibility	0.399(0,053)	0.157 (NS)	0,26 (0.025*)	-0.4 (<0.001*)
	Total	0.466(0.022*)	0.211 (NS)	-0.38 (0.015*)	0.16(NS)
**SRS-22**	Function	0.035(NS)	-0.008 (NS)	-0.173 (NS)	0.057 (NS)
	Pain	0.161 (NS)	0.376(NS)	-0.057(NS)	0.13(NS)
	Body image	0.851 (NS)	-0.178 (NS)	-0.05 (NS)	-0.11 (NS)
	Psychosocial status	-0.083 (NS)	0.242(NS)	0,29(0.01*)	-0.16 (NS)
	Satisfaction	0.159 (NS)	0.010 (NS)	0.26 (0,025*)	0.4 (0.01*)
	Total	1.98(NS)	0.224(NS)	0.27(0.002*)	-0.27(NS)

QLPSD: Quality of Life Profile for Spine Deformities" (QLPSD) scale, SRS-22: The Scoliosis Research Society 22 questionnaire; visual analogue scale objectifying the QOL (EVA-QOL); *: p<0,05; NS: nonsignificant

According to the QLPSD scale, our results identified a correlation between QOL measured and age (as age increases, overall QLPSD score also increases; r=0.466; p=0.022). Furthermore, we noted that a higher percentage of correction was associated with a better psychosocial state, an improvement in body image, and greater satisfaction with QOL (as measured by the QLPSD). These associations were found to be statistically significant with correlation coefficients (r) of -0.23, -0.28 and -0.38 respectively. We also noticed that a longer period of treatment was linked to enhancements in both the psychosocial condition (r=-0.25, p=0.031) and dorsal flexibility (r=-0.4, p<0.001) in adolescents. On the other hand, we found that no correlation was observed between sleep disorders, back pain, and the studied factors and no significant correlation between the Risser index and the different domains of the QLPSD (Table 3).

According to the SRS-22, the overall QOL score of patients wearing the brace was significantly correlated with the percentage of correction by brace. In addition, we noted a correlation between the psychosocial state and the percentage of correction. Satisfaction domain was correlated with the percentage of correction and the treatment duration (r= 0.26, p= 0.02 and r=0.4, p=0.01; respectively). It means that the satisfaction increased with the correction percentage and with the treatment duration. However, no correlation was observed between all SRS-22 domains and age or Risser index (Table 3).

## Discussion

Based on our findings, it appears that the quality of life of adolescents with idiopathic scoliosis who were treated with a brace was significantly decreased compared to those who only underwent rehabilitation. This conclusion was drawn from the results obtained from the three quality of life scales that were used in the study. These findings indicate that even though wearing a brace for idiopathic scoliosis treatment has benefits such as being lightweight, discreet under clothing, causing minimal discomfort during daily activities, and can be manufactured on an outpatient basis, it still results in a significant alteration of the patient's quality of life. It is noteworthy that this alteration occurs despite the advantages mentioned earlier, including the fact that it does not require school absenteeism or cause the patient to be estranged from their family environment [17]. Studies in the past have produced conflicting results regarding the impact of bracing on QOL. Some papers including a meta-analysis have reported no decrease in QOL in patients with AIS who received brace treatment [18-20]. For instance, Olafsson found that bracing did not have a negative impact on the self-image of adolescent patients [19]. While it was noted that bracing may reduce self-esteem at the beginning of treatment, there were no significant differences in the rate of psychopathologies between brace-treated patients and healthy individuals of the same age [21]. Furthermore, Noonan and colleagues found slight differences in the psychological well-being of scoliotic adolescents who received bracing compared to healthy controls. These differences tended to disappear in adulthood [22].

On the other hand, other investigations including a review have shown that the brace constitutes a psychological obstacle with deleterious effects on QOL [23,24]. Indeed, some authors reported brace treatment to be associated with high levels of stress and poor QOL [25]. For instance, a study conducted by Freidel and colleagues found that patients treated with braces for AIS had a high prevalence of depressed mood and reduced QOL [23]. Additionally, bracing has been associated with negative body image, reduced self-esteem, increased stress, and a higher likelihood of developing phobias and anxiety compared to surgical treatment [26]. Notably, Kotwicki *et al*. discovered that stress levels were higher in AIS patients when they were asked about their braces instead of their deformities [27]. Our results are consistent with many other studies that have assessed various types of braces [28,29].

Possible explanations for these diverging results may be attributed to variations in the instrument used to measure outcomes, brace features, treatment duration, compliance to treatment, or cultural disparities. Our results suggested that the QOL measured by QLPSD decreased with age. Similarly, a recent research showed that participants under the age of 13 had a superior self-image score on the SRS-22 than adolescents beyond the age of 13 [30]. The higher brace compliance in younger than in older teenagers may be a possible reason for these findings. In our study, we found that as the percentage of correction increased, the QOL measured by EVA-QOL improved. We also observed a correlation between the percentage of correction by the brace and various QOL domains. Our results were consistent with other studies [15]. In addition, we found a correlation between the treatment duration and both domains related to QOL (psychosocial condition and dorsal flexibility). This could be explained by the adaptation of adolescents to wearing the brace over time, as well as the positive effect of progress made during treatment and follow-up [11]. Furthermore, our results stipulated that the longer the duration of treatment, the greater the satisfaction measured by SRS-22. One possible explanation for this is that as adolescents grow older, they become more cognizant and have higher expectations of their social life, which can boost their level of satisfaction. This outcome is consistent with Babaee *et al*. findings, which indicated that while early brace treatment can have a positive effect on self-esteem and psychological well-being in adolescents with AIS, the initial adaptation period may pose some challenges. However, once patients get used to wearing the brace, these difficulties tend to go away [31].

Finally, the overall QOL score evaluated by QLPSD patients with ASI in our study was not correlated with the Risser index, unlike the study conducted by Climent *et al*. showing that bone maturation was significantly correlated with QOL [15]. Our work had some limitations. In fact, the sample size was relatively small. Additionally, our two groups are significantly different in terms of Cobb angle. This is explained by the fact that they do not have the same indications. On the other hand, although the QLPSD includes items suitable for children aged 10 to 20, this questionnaire does not consider important areas of adolescent life, such as sports activities, schooling, treatment compliance and the role of parents in accepting the disease and its management.

## Conclusion

The brace treatment had a negative impact on QOL domains according to the three rating scales used in this study. This would influence adherence to treatment in adolescents mainly by reducing the duration of wearing per day. Further studies should examine how this QOL varies over time in this adolescent population. Additional considerations must be made as part of a multidisciplinary team: physicians, physiotherapists, ergotherapists and orthoprosthesists for the brace design, and systematic psychological support must be offered to the children and their family in order for them to fully accept the suggested treatment.

### What is known about this topic

- Findings are currently inconclusive about the impact of different treatments on the QOL among adolescents with AIS;
- Self-image and mental health were the most frequently reported affected domains.

### What this study adds

- This study contributes to the existing literature by assessing the QOL of patients with AIS in a developing country;
- Brace decreased significantly all QOL domains among patients with AIS in comparison to those who underwent rehabilitation without the use of a brace;
- Age and treatment duration were found to have a significant relationship with QOL in adolescents with AIS.
